# Baseline tumor burden and outcomes in patients with rare cancers treated with immunotherapy (Southwest Oncology Group trial S1609)

**DOI:** 10.1002/cncr.70374

**Published:** 2026-03-27

**Authors:** Paul L. Swiecicki, Megan Othus, Sandip P. Patel, Young Kwang‐Chae, Razelle Kurzrock

**Affiliations:** ^1^ University of Michigan Rogel Cancer Center Ann Arbor Michigan USA; ^2^ Southwest Oncology Group Cancer Research Network Statistical Center Seattle Washington USA; ^3^ Fred Hutchinson Cancer Center Seattle Washington USA; ^4^ University of California at San Diego Moores Cancer Center La Jolla California USA; ^5^ Feinberg School of Medicine Northwestern University Chicago Illinois USA; ^6^ Medical College of Wisconsin Milwaukee Wisconsin USA

**Keywords:** immune checkpoint inhibitor, immunotherapy, predictive biomarkers, rare cancers, Response Evaluation Criteria in Solid Tumors (RECIST), Southwest Oncology Group study S1609, tumor burden

## Abstract

**Background:**

It has been suggested that baseline tumor burden may correlate with immune checkpoint inhibitor (ICI) outcome for individual tumor types in which ICIs are standardly used. The authors investigated whether pretreatment tumor burden correlates with overall survival (OS), progression‐free survival (PFS), and tumor regression among patients who had rare cancers treated with dual ICIs.

**Methods:**

Southwest Oncology Group study S1609 was a phase 2, National Cancer Institute/Southwest Oncology Group basket study (>1000 sites) evaluating nivolumab plus ipilimumab in 53 cohorts of patients who had rare/ultrarare malignancies (ClinicalTrials.gov identifier NCT02834013). Overall, 722 patients were included in this secondary analysis, all of whom had measurable disease (Response Evaluation Criteria in Solid Tumors, version 1.1). Baseline tumor burden, defined as the sum of the greatest dimensions of target lesions at study registration, was analyzed based on quartiles observed in the data. The number of target lesions was also considered a secondary tumor burden measure. End points included OS and PFS.

**Results:**

Larger baseline tumor burden correlated with shorter OS, but not PFS (multivariable analysis). Higher baseline tumor burden quartiles had only a weak negative association with any tumor regression at first scan (Fisher exact test, *p* = .09), and multivariable analyses further indicated that both tumor burden and any tumor regression at first posttreatment scan were independently associated with OS in multivariable analysis (comparing a baseline tumor size ≥12.9 cm vs. 1.0–4.8 cm; hazard ratio, 1.64; 95% confidence interval, 1.02–1.72; *p* = .032), but there was no evidence of an interaction between tumor burden and any tumor regression at the first scan (*p* for interaction > .65).

**Conclusions:**

Larger baseline tumor burden was associated with worse OS, but not PFS, and was not predictive of tumor regression after dual ICI therapy in a large cohort with rare cancer types.

## Abstract 2

Higher tumor burden, as measured by standard‐of‐care imaging, has been implicated as a marker for poor outcome after immune checkpoint inhibitor therapy for individual common cancer types in which immune checkpoint blockade is routinely used. This secondary analysis of 722 evaluable patients enrolled in a large, prospective, National Cancer Institute, multibasket trial evaluating nivolumab plus ipilimumab demonstrates that larger baseline tumor burden, as assessed by tumor size or by the number of target lesions, correlates significantly and independently with shorter survival but is not similarly associated with progression‐free survival or tumor regression.

## INTRODUCTION

The advent of immune checkpoint inhibitors (ICIs) has revolutionized the management of many solid tumors, resulting in new treatment paradigms, less treatment‐related toxicity, and improved survival. Despite this excitement, ICIs only provide benefit in a minority of patients and can result in significant side effects, which may be life‐threatening. Predictive treatment biomarkers enable physicians to better choose therapy. Programmed death‐ligand 1 (PD‐L1) expression detected by immunohistochemistry is an inconsistent biomarker, with predictive capacity in only some cancers.^1^, ^2^ Furthermore, technical issues challenge its use, including variable cutoffs for different diseases/ICIs, variable performance of PD‐L1–antibody clones, and poor interrater reliability.^3^ Notably, the correlation between predictive biomarkers and ICI use across rare cancers is poorly studied. Rare cancers are often excluded from large clinical trials, have limited approved therapeutic options, and treatment is often based on small case series.

There is a biologic rationale to support a relation between patient outcome after ICIs and tumor burden. Preclinical data from numerous cancer models suggest that larger tumors are more immunosuppressive compared with smaller volume tumors, possibly because of various mechanisms, including myeloid‐derived suppressor cell infiltration, tumor‐associated macrophages, and immunosuppressive cytokine production.^4^, ^5^, ^6^, ^7^, ^8^

Anatomic (computed tomographic or magnetic resonance) imaging is obtained routinely as the standard of care. Radiographic measurements are an easily accessible assessment of tumor volume. We hypothesized that patients with smaller volume tumors at baseline would have longer survival when treated with dual ICI therapy

## MATERIALS AND METHODS

### Patients and procedures

Southwest Oncology Group study SWOG 1609 (DART) was an open‐label, National Cancer Institute‐supported, phase 2 basket study that evaluated the combination of nivolumab and ipilimumab in rare malignancies; it included 53 baskets (ClinicalTrials.gov identifier NCT02834013). Study medication was provided by Bristol Myers Squibb Company through the National Cancer Institute Cancer Therapy Evaluation Program. The study was conducted in accordance with the Declaration of Helsinki, and all participants provided informed consent.

All patients had measurable disease according to Response Evaluation Criteria in Solid Tumors, version 1.1 (RECIST v1.1), and patients must have had advanced, rare cancers with no other treatment options available that demonstrated the ability to prolong overall survival (i.e., in a randomized trial against another standard treatment or in a comparison with historical controls). Patients who could not receive other standard therapy that demonstrated the ability to prolong survival because of medical issues were eligible if other eligibility criteria were met.

Patient race and ethnicity were collected and self‐reported. RECIST v1.1 prompts clinicians to define target lesions that are chosen based on their size, reproducibility, and extent to involve multiple organs (if pertinent). Up to five target lesions are chosen at baseline, and the baseline sum of greatest dimensions is used as the reference of overall tumor burden.^9^

Patients were treated with nivolumab 240 mg every 2 weeks and ipilimumab 1 mg/kg every 6 weeks, both intravenously. Response assessments were performed at baseline; at weeks 8, 16, and 24; then every 12 weeks. Response was assessed locally by RECIST v1.1 criteria; central review or submission of imaging was not included in the trial design. Treatment was continued until tumor progression, unacceptable toxicity, or withdrawal of consent.

### End point definitions, assessment of baseline tumor burden, outcome, and statistical associations

All eligible patients who received at least one dose of protocol therapy (eligible and evaluable patients per protocol). Overall survival (OS) was measured from the date of treatment start to the date of death from any cause, with patients last known to be alive censored at the date of last contact. Progression‐free survival (PFS) was measured from the date of treatment start to the first date of cancer progression according to RECIST v1.1 or death from any cause, with patients last known to be alive without progression censored at the date of last contact.

Baseline tumor burden was defined as the sum of the greatest dimensions of target lesions according to RECIST v1.1 at the time of study registration; the number of target lesions was assessed as a secondary measure of tumor burden.

OS and PFS were estimated using the Kaplan–Meier method; 54 patients were censored for the PFS end point, and 143 were censored for the OS end point. Associations with OS and PFS were assessed with Cox regression models, which were also stratified by basket (see Table S1) to account for various hazards across the baskets. Martingale residuals from Cox regression models with no covariates were plotted against baseline tumor burden to visually evaluate patterns in association. P‐spline models were used to formally evaluate evidence of nonlinearity. Multivariable models were completed and case controlled for baseline age, sex, and Eastern Cooperative Oncology Group performance status regardless of univariate significance. Association between baseline tumor burden and any tumor regression at the first scan was also evaluated using the Fisher exact test; any tumor regression was defined based on change in tumor burden decreasing by any amount between baseline and the first scan. Additional analyses of OS and PFS assessing associations with tumor regression at the first scan were landmarked at day 65 (the first scan was scheduled according to protocol at 8 weeks after trial registration; data on scans were provided on the trial case report form); participants who had progressed or died before day 65 were exclude from this additional analysis. Frailty models (with basket; see Table S1) are reported as a sensitivity analysis. Analyses were conducted with R version 4.5.2 (R Foundation for Statistical Computing). Two‐sided alpha of 5% was used to interpret statistical significance.

## RESULTS

### Patient characteristics

Altogether, 722 evaluable patients were included in this analysis. The median follow‐up for patients who were alive at last contact was 4.1 years. Demographics and tumor characteristics are listed in Table 1. The median patient age was 60 years (range, 18–88 years); 52% of patients were women, 55% had an Eastern Cooperative Oncology Group performance status of 1 at enrollment, most patients identified as White. The median follow‐up at the time of this analysis was 49 months (range, 1–77 months). Enrollment into study baskets and the numbers patients included in this analysis by basket are listed in Table S1.

**TABLE 1:** Characteristics of 722 evaluable patients included in analyses.^a^

		Baseline tumor burden quartiles^a^				
Characteristic	Entire cohort, *n* = 722	1.0–4.7 cm, *n* = 188	4.8–8.0 cm, *n* = 176	8.1–12.8 cm, *n* = 180	≥12.0 cm, *n* = 178	*p*
Age at study registration: Median [range], years	60 [18–88]	63 [18–88]	60 26–84]	60 [19–87]	58 [20–85]	.018^b^
Sex						.94
Female	378 (52.0)	98 (52.0)	95 (54.0)	95 (53.0)	90 (51.0)	
Male	344 (48.0)	90 (48.0)	81 (46.0)	85 (47.0)	88 (49.0)	
Zubrod performance status						.034^b^
0	278 (39.0)	77 (41.0)	73 (41.0)	75 (42.0)	53 (30.0)	
1	399 (55.0)	103 (55.0)	95 (54.0)	96 (53.0)	105 (59.0)	
2	45 (6.0)	8 (4.0)	8 (5.0)	9 (5.0)	20 (11.0)	
Race						.37
Asian	27 (4.0)	7 (4.0)	10 (6.0)	5 (3.0)	5 (3.0)	
Black	73 (10.0)	17 (9.0)	10 (6.0)	23 (13.0)	23 (13.0)	
Native American or Alaskan	6 (1.0)	1 (1.0)	1 (1.0)	3 (2.0)	1 (1.0)	
Native Hawaiian or Pacific Islander	2 (<1.0)	0 (0.0)	2 (1.0)	0 (0.0)	0 (0.0)	
White	575 (80.0)	154 (82.0)	144 (82.0)	138 (77.0)	139 (78.0)	
More than one race	1 (<1.0)	0 (0.0)	0 (0.0)	0 (0.0)	1 (1.0)	
Not reported	38 (5.0)	9 (5.0)	9 (5.0)	11 (6.0)	9 (5.0)	
Hispanic						.56
Yes	62 (9.0)	12 (6.0)	16 (9.0)	19 (11.0)	15 (8.0)	
No	660 (9.01)	176 (94.0)	160 (91.0)	161 (89.0)	163 (92.0)	
No. of target lesions						
Median no. [range]	2 [1–5]	1.5 [1–3]	2 [1–5]	3 [1–5]	4 [1–5]	< .001^b^
1		94 (50.0)	27 (15.0)	16 (9.0)	11 (6.0)	< .001^b^
2		87 (46.0)	87 (49.0)	44 (24.0)	23 (13.0)	
3		7 (4.0)	45 (26.0)	55 (31.0)	33 (19.0)	
4		0 (0.0)	16 (9.0)	46 (26.0)	53 (30.0)	
5		0 (0.0)	1 (1.0)	19 (11.0)	58 (33.0)	
No. of nontarget lesions						
Median no. [range]	2 [0–10]	1 [0–8]	1 [0–5]	2 [0–10]	2 [0–10]	.011^b^
0		46 (24.0)	53 (30.0)	32 (18.0)	50 (28.0)	.003^b^
1		54 (29.0)	44 (25.0)	40 (22.0)	31 (17.0)	
2		44 (23.0)	35 (20.0)	51 (28.0)	36 (20.0)	
3		15 (8.0)	22 (12.0)	24 (13)	18 (10.0)	
4		14 (7.0)	15 (9.0)	11 (6)	15 (8.0)	
≥5		15 (8.0)	7 (4.0)	22 (12)	28 (16.0)	
Tumor change at first on‐treatment scan						
Did not receive first scan	78 (11.0)	22 (12.0)	13 (7.0)	15 (8.0)	28 (16.0)	.09
Any tumor regression	167 (23.0)	50 (27.0)	44 (25.0)	41 (23.0)	32 (18.0)	
No tumor regression	477 (66.0)	116 (62.0)	119 (68.0)	124 (69.0)	118 (66.0)	

^a^ Except for age and the number of lesions, numbers represent the number of patients (%); baseline tumor burden was based on the size of target lesions.

^b^ These *p* values indicate a statistically significant difference.

### Outcomes versus baseline tumor burden

**FIGURE 1:**
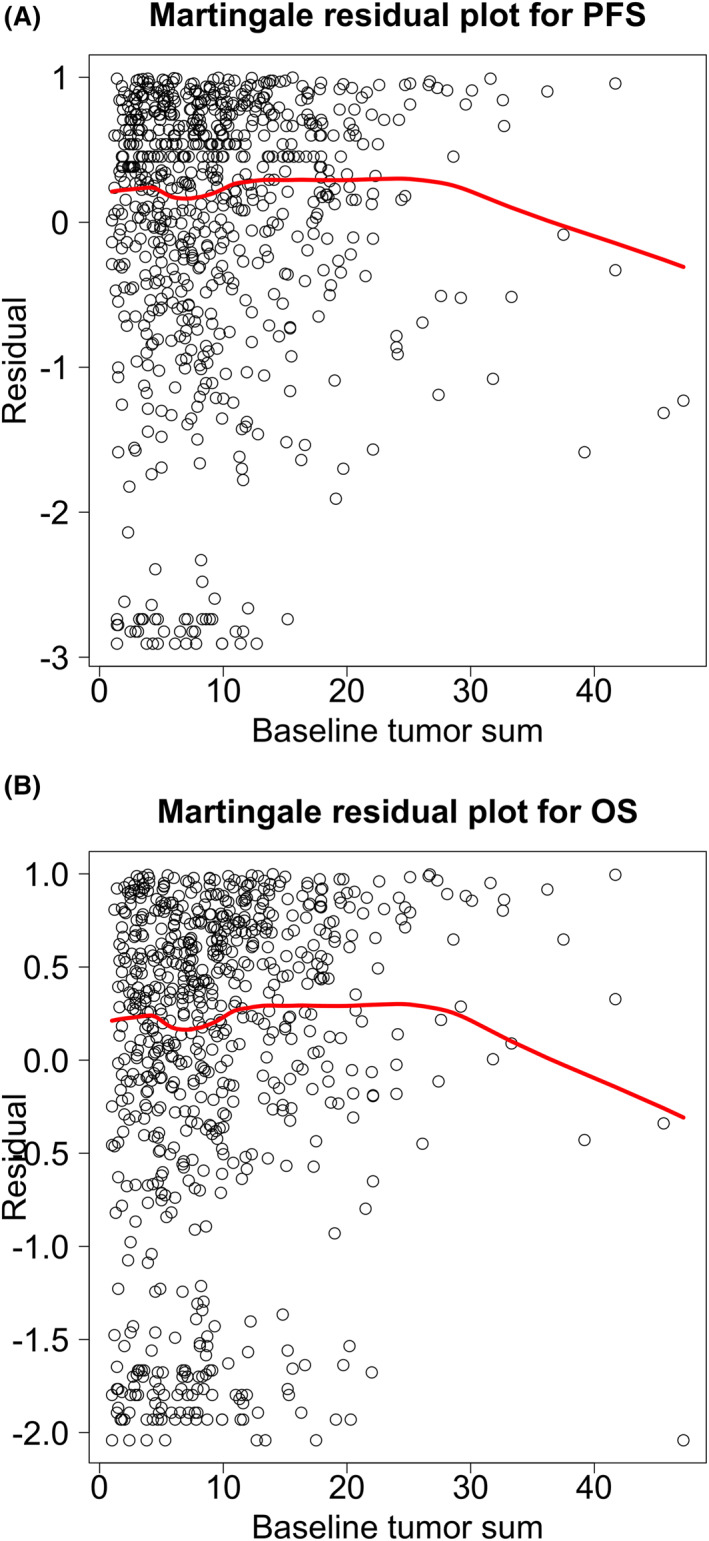
(A,B) Martingale residual plots for PFS and OS. For each patient, their residual is equal to the difference between their event indicator (equal to 0 if censored, equal to 1 otherwise) and the probability of an event at their censoring or event time (probability based on calculated cumulative incidence). The largest value the residual can take is 1. Censored patients with long follow‐up will have more negative residuals. The spline estimate of the pattern of association between baseline tumor measurement and the residual is indicated by the red curve. These plots illustrate that larger values of baseline tumor sum are associated with shorter OS, and the association between baseline tumor sum and PFS and OS may not be linear because the red lines have nonlinear features. OS indicates overall survival; PFS, progression‐free survival.

We initially evaluated Martingale residual plots for baseline tumor size (median, 8 cm; 25th percentile, 4.7 cm; 75th percentile, 12.8 cm) versus both PFS and OS (Figure 1A,B), with a larger residual corresponding to a higher risk of progression and death, respectively. Martingale residual plots help assess the shape of an association between a quantitative covariate and a survival model when dealing with censored data (which are identified from PFS or OS data). In this cohort, the Martingale residual plots for PFS were relatively flat across baseline tumor sizes. The plots for OS indicated a generally increasing trend in the risk of death with increasing baseline tumor burden, with a flattening of risk at larger sizes. P‐spline regression models indicated similar conclusions as the Martingale residual plots; the *p* value for a test of nonlinearity with PFS was *p* = .12; and, for a test of nonlinearity with OS, it was *p* = .004.

**FIGURE 2:**
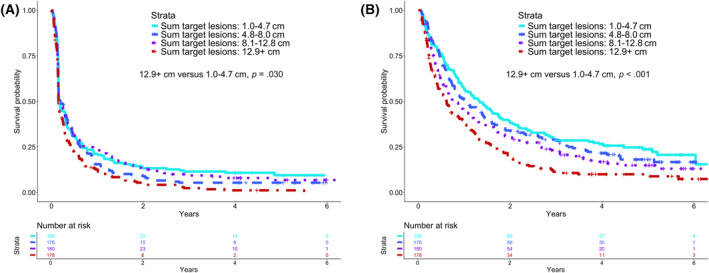
(A) Kaplan–Meier estimates and Cox regression summaries of progression‐free survival by baseline tumor size quartile. Cox models were stratified by histologic basket. Multivariable models were completed and case controlled for baseline age, sex, and Eastern Cooperative Oncology Group performance status regardless of univariate significance. (B) Kaplan–Meier estimates and Cox regression model summaries of overall survival by baseline tumor size quartile. Cox models were stratified by histologic basket. Multivariable models were completed and case controlled for baseline age, sex, and Eastern Cooperative Oncology Group performance status regardless of univariate significance.

To capture potentially nonlinear trends in the baseline tumor size data, we also evaluated baseline tumor burden (as defined by the sum of greatest dimensions) based on quartiles observed in the data (1.0–4.7, 4.8–8.0, 8.1–12.8, and ≥12.9 cm). Tests of the proportional hazards assumption indicated that the four‐group version of this variable did not violate the assumption (PFS, *p* = .22; OS, *p* = .20). Kaplan–Meier survival estimates of PFS and OS for baseline tumor burden quartiles are illustrated in Figure 2A,B along with results from univariate and multivariable Cox regression models. The two largest quartiles of tumor burden size had significantly worse OS compared with the first (smallest) quartile (multivariable hazard ratio [HR], 1.64; 95% confidence interval [CI], 1.02–1.72; *p* < .001). There was no significant association between quartile size and PFS in a multivariable model. Conclusions remained the same when evaluating frailty models as a sensitivity analysis (see Table S2).

We observed similar patterns when evaluating an alternative measure of tumor burden, the number of target lesions (see Figure S1A,B). Participants who had four and five target lesions had significantly shorter OS than participants who had one target lesion on multivariable analysis (four lesions: HR, 1.52 [95% CI, 1.13–2.04; *p* < .001]; five lesions: HR, 1.78 [95% CI, 1.27–2.48; *p* < .001]). The number of target and nontarget lesions was correlated with tumor burden as measured by baseline target lesion quartiles (Table 1).

Finally, given the indication of worse survival among patients in the higher baseline tumor burden quartiles, we wanted to assess whether the survival differences were caused by the trial therapy versus the underlying natural history of the disease (i.e., did tumor burden not predict tumor response to therapy or was it prognostic of the overall outcome). A Fisher exact test comparing higher baseline tumor burden quartiles and any tumor regression at first scan had a *p* value of .09 (Table 1).

In regression analyses, both tumor burden (measured by tumor burden quartiles and by the number of target lesions) and any tumor regression at the first posttreatment scan were independently associated with OS in univariate and multivariable analysis (see Tables S3 and S4), but there was no evidence of an interaction between tumor burden and any tumor regression at the first scan (*p* for interaction > .65).

## CONCLUSION

Despite rapid advances in immunotherapeutic treatment approaches for solid tumors, limited biomarkers exist to identify the likelihood of treatment efficacy and outcome. Commonly used pan‐cancer predictive biomarkers, such as high tumor mutational burden evaluated by next‐generation sequencing, are relatively uncommon, occurring in 13%–26% of solid tumors.^10^, ^11^ Other biomarkers, such as tumor PD‐L1 expression by immunohistochemistry, have variable predictive ability.^12^ Therefore, we sought to examine the relation between an easily available parameter—baseline tumor burden, as reflected by size of or number of target lesions on imaging—and outcome parameters, including PFS, OS, and tumor regression.

Taken together, our data suggest that, in a large cohort of diverse, rare cancer tumor types treated prospectively with nivolumab and ipilimumab, higher tumor burden (as characterized by either the size of or the number of target lesions) correlates significantly and independently with shorter OS; however, correlation with PFS was not significant in multivariable analysis. Although previous studies have investigated the relation between imaging‐assessed tumor burden and outcomes with ICIs, to the best of our knowledge, this is the first study evaluating their performance in rare cancers. These previous studies specifically assessed single tumor types in which ICIs are an approved therapy, including head and neck cancers, nonsmall cell lung cancer, and melanoma.^13^, ^14^, ^15^, ^16^, ^17^ Although various analyses and cutoff points for tumor burden have been used, these data have also consistently demonstrated that larger baseline tumor burden is associated with worsened outcomes, such as shorter OS.^13^, ^15^, ^17^ Baseline fludeoxyglucose‐18–positron emission tomographic/computed tomographic imaging, which also reflects metabolic activity, also predicts outcomes in a similar manner.^18^

Further work is needed to define better predictive biomarkers for immunotherapy benefit that can be applied across diverse cancers in a cost‐effective fashion using commonly available patient resources. Although some tissue‐based or blood‐based molecular tests have demonstrated early promise of predicting response to ICI, their role in predicting ICI outcomes remains complicated.^19^, ^20^ Imaging is a tool that is broadly used and thus is amenable to analysis in relation to outcome. However, one of the challenges of these studies is to determine whether larger tumor burden is related directly to poorer OS after ICI therapy or is simply a prognostic factor that predicts worse outcome regardless of therapy. Indeed, it is known that larger tumor burdens generally correlate with worse outcomes in patients with cancer across treatments.^21^ One way to study this issue is to examine the correlation between tumor burden and tumor regression after ICI treatment, because it would be assumed that regression was related to ICI therapy. Our study suggests that tumor burden may correlate with outcome as a prognostic factor—meaning that, in general, patients with larger tumor burdens do worse—rather than as a specific factor related to immunotherapy. Evidence for this assertion lies in our observation that higher baseline tumor burden quartiles had only a weak (nonsignificant; *p* = .09) negative correlation with any tumor regression at the first scan. Moreover, multivariable analyses indicated that both tumor burden (measured by tumor burden quartiles and by the number of target lesions) and any tumor regression at the first posttreatment scan were independently associated with OS in multivariable analysis, and there was no evidence of an interaction between tumor burden and any tumor regression at the first scan (*p* for interaction > 0.65). That said, interaction tests notoriously are of low power, and this trial was not prospectively designed or powered to evaluate this interaction test.

The finding of a significant association with OS but not PFS could be attributed to several factors. In this high‐risk patient population, many patients progressed during the first 3–4 months of therapy, as indicated by a sharp drop in the PFS curves in Figure 2. In addition, the protocol‐dictated scan schedule limited and harmonized the times at which progression was evaluated. Also, OS captures the impact of subsequent therapies, whereas PFS does not. The limited data collection in this publicly funded trial did not allow us to collect and capture these data for the purpose of a formal evaluation.

A limitation of this study is that‐cross sectional imaging (e.g., computed tomography or magnetic resonance imaging) is one‐dimensional and does not capture the volume of tumors. Furthermore, although RECIST is an invaluable tool to standardize radiographic response assessment in clinical trials, only some of a patient’s metastatic deposits may be selected as target lesions. In addition, target lesions may not even be the largest metastases, and there is no field in which the absolute number of metastases is recorded. Therefore, the *sum of greatest dimensions* may not fully capture the volume or extent of disease.^9^ Other limitations pertain to the many types of cancers in this trial. Although the diversity of rare cancers included in this large study is a novel addition to the literature and may suggest generalizability of the observations across cancer types, the limited number of patients with each tumor type and basket (see Table S1), as well as the limited number of objective responses in each tumor type, precludes analyzing the role of these evaluations in individual rare histologies. The latter should be a subject of future investigations. This publicly funded trial had limitations on data collection resources, thus multivariable models were only able to control for the limited variables that were collected across all baskets.

In summary, among a large cohort of patients who had rare solid tumors treated with dual ICIs, traditionally used tumor measurement characteristics, such as the size and number of target lesions, correlated with shorter OS but did not predict the degree of tumor regression (a direct measure of ICI action). Further studies are needed to define readily usable, cost‐effective biomarkers to guide the use of immunotherapy among diverse cancers.

## AUTHOR CONTRIBUTIONS

**Paul L. Swiecicki:** Conceptualization; data curation; investigation; formal analysis; writing—original draft; writing—review and editing; and project administration. **Megan Othus:** Conceptualization; methodology; data curation; investigation; validation; formal analysis; writing—original draft; writing—review and editing; and resources. **Sandip P. Patel:** Investigation; writing—review and editing; funding acquisition; resources; and project administration. **Young Kwang‐Chae:** Investigation; writing—review and editing; funding acquisition; resources; and project administration. **Razelle Kurzrock:** Conceptualization; methodology; investigation; validation; formal analysis; writing—original draft; writing—review and editing; project administration; supervision; funding acquisition; and resources.

## CONFLICT OF INTEREST STATEMENT

Paul L. Swiecicki reports personal/consulting fees from CDR Life, EMD Cerano, GeoVax, Janssen, Rapt, Regeneron Pharmaceuticals, Remix, and Rgenta; and support for other professional activities from Astellas Pharma, Elevar, and Prelude Therapeutics outside the submitted work. Megan Othus reports personal/consulting fees from Biosight, Bristol Myers Squibb Company, Merck, and Refined Oncology; and data and safety monitoring for Celgene Corporation, Glycomimetics, and Grifols Therapeutics Inc. outside the submitted work. Sandip P. Patel reports institutional grants/contracts from Amgen, AstraZeneca/MedImmune, A2bio, Bristol Myers Squibb Company, Eli Lilly and Company, Fate Therapeutics, Gilead, Iovance, Merck, Pfizer, Roche/Genentech, and Tscan; and personal/consulting or advisory fees from Amgen, AstraZeneca, BeiGene, Bristol Myers Squibb Company, Certis, Daiichi‐Sankyo Company, Eli Lilly and Company, Genentech, Gilead, Illumina, Jazz Pharmaceutical, Johnson & Johnson, Merck, Pfizer, Signateria, and Tempus outside the submitted work. Young Kwang‐Chae reports grants or contracts from AbbVie, Biodesix, Bristol Myers Squibb Company, Freenome, Imagene AI, Oncohost, Picture Health, Predicine, Regeneron, and Tempus; and payment or honoraria for lectures, presentations, or speakers' bureaus from AstraZeneca, Biodesix, Boehringer Ingelheim, Bristol Myers Squibb Company, Esai, Foundation Medicine, GeneCker, Guardant Health, Immuneoncia, Jazz Pharmaceutical, Lilly Oncology, Lunit, Merck, Neogenomics, NeoImmunTech, Novocure, Oncohost, Picture Health, Regeneron, Roche/Genentech, Takeda, and Tempus outside the submitted work. Razelle Kurzrock reports research funding from Boehringer Ingelheim, Debiopharm, Foundation Medicine, Genentech, Grifols, Guardant, Incyte, Konica Minolta, Medimmune, Merck Serono, Omniseq, Pfizer, Sequenom, Sysmex, Takeda, and TopAlliance; institutional research funding from the National Cancer Institute; personal/consulting or advisory fees from Actuate Therapeutics, AstraZeneca, Bicara Therapeutics Inc., Biological Dynamics, Caris, Daiichi‐Sankyo Company, Datar Cancer Genetics, EISAI, EMD Serono, EOM Pharmaceuticals, Iylon, Jackson Laboratories, LabCorp, Lanauria Therapeutics, Merck, NeoGenomics, Neomed, Pfizer, Precirix, Prosperdtx, Quanta Therapeutics, Recordati, Regeneron, Roche, TD2/Volastra, Turning Point Therapeutics, X‐Biotech; support for professional activities from the Worldwide Innovative Network (WIN Consortium) for Personalized Cancer Therapy; serves on the boards of CureMatch, CureMetrix, and XZOM; and is a co‐founder of CureMatch Inc. and owns an equity interest in the company outside the submitted work.

## Supporting information

Supplementary Material

## Data Availability

The data analyzed in this study are available upon request following Southwest Oncology Group policies and procedures (https://www.swog.org/sites/default/files/docs/2019‐12/Policy43_0.pdf).
