# Supplementary material for: Baseline tumor burden and outcomes in patients with rare cancers treated with immunotherapy (Southwest Oncology Group trial S1609)
Source: Cancer. 2026 Mar 27;132(7):e70374. doi: 10.1002/cncr.70374 (PMC13025072; doi:10.1002/cncr.70374)
Supplement: Supplementary file 1 — Supplementary Material [file CNCR-132-e70374-s001.docx]

**Table S1: Histologic baskets and sample size from the S1609 DART clinical trial and the data evaluable in the current analysis**

|  | N enrolled | N in the analysis |  |
| --- | --- | --- | --- |
| Basket 1: Epithelial tumors of nasal cavity, sinuses, nasopharynx | | 7 | 7 |
| Basket 2: Epithelial tumors of major salivary glands | | 30 | 29 |
| Basket 3: Salivary gland type tumors of head, neck, lip, espophagus, stomach, trachea and lung, breast, and other locations. | | 6 | 5 |
| Basket 4: Undifferentiated carcinoma of gastrointestinal (GI) tract | | 6 | 6 |
| Basket 5: Adenocarcinoma with variants of small intestine | | 26 | 24 |
| Basket 6: Squamous cell carcinoma with variants of GI tract | | 6 | 6 |
| Basket 7: Fibromixoma, low grade mucinous adenocarcinoma (appendix,ovary) | | 10 | 10 |
| Basket 8: Rare Pancreatic tumors | | 11 | 7 |
| Basket 9: Intrahepatic cholangiocarcinoma | | 9 | 9 |
| Basket 10: Extrahepatic cholangiocarcinoma and bile duct tumors | | 10 | 10 |
| Basket 11: Sarcomatoid carcinoma of lung | | 16 | 13 |
| Basket 12: Bronchoalveolar carcinoma lung | | 8 | 8 |
| Basket 13: Non-epithelial tumors of the ovary | | 17 | 17 |
| Basket 14: Trophoblastic tumor | | 3 | 3 |
| Basket 15: Transitional cell carcinoma (not renal, pelvis, ureter, bladder) | | 1 | 1 |
| Basket 16: Cell tumor of the testes and extragonadal germ tumors | | 17 | 17 |
| Basket 17: Epithelial tumors of penis | | 23 | 23 |
| Basket 18: Squamous cell carcinoma variants of the genitourinary (GU) system | | 7 | 6 |
| Basket 19: Spindle cell carcinoma of kidney, pelvis, ureter | | 10 | 10 |
| Basket 20: Adenocarcinoma with variants of GU system (not prostate) | | 9 | 9 |
| Basket 21: Odontogenic malignant tumors | | 4 | 4 |
| Basket 22: Endocrine carcinoma of pancreas and digestive tract | | 22 | 19 |
| Basket 23: Neuroendocrine carcinoma including carcinoid of the lung | | 35 | 32 |
| Basket 24: Pheochromocytoma, malignant | | 7 | 7 |
| Basket 25: Paraganglioma | | 6 | 6 |
| Basket 26: Carcinomas of pituitary, thyroid, parathyroid, adrenal cortex | | 21 | 19 |
| Basket 27: Desmoid tumors | | 17 | 16 |
| Basket 28: Peripheral nerve sheath tumors and NF1-related tumors | | 9 | 8 |
| Basket 29: Malignant giant cell tumors | | 6 | 6 |
| Basket 30: Chordoma | | 11 | 10 |
| Basket 31: Adrenal cortical tumors | | 23 | 21 |
| Basket 32: Tumor of unknown primary (Cancer of Unknown Primary; CuP) | | 26 | 21 |
| Basket 33: Not Otherwise Categorized (NOC) Rare Tumors | | 65 | 62 |
| Basket 34: Adenoid cystic carcinoma | | 29 | 26 |
| Basket 35: Vulvar cancer | | 17 | 16 |
| Basket 36: MetaPLASTIC carcinoma (of the breast) | | 19 | 17 |
| Basket 37: Gastrointestinal stromal tumor (GIST) | | 12 | 11 |
| Basket 38: Perivascular epithelioid cell tumor (PEComa) | | 17 | 16 |
| Basket 39: Apocrine tumors/Extramammary Paget‚Äôs Disease | | 9 | 9 |
| Basket 40: Peritoneal mesothelioma | | 18 | 17 |
| Basket 41: Basal cell carcinoma | | 18 | 17 |
| Basket 42: Clear cell cervical cancer | | 5 | 5 |
| Basket 43: Esthenioneuroblastoma | | 7 | 6 |
| Basket 44: Endometrial carcinosarcoma (malignant mixed Mullerian tumors) | | 25 | 22 |
| Basket 45: Clear cell endometrial cancer | | 9 | 8 |
| Basket 46: Clear cell ovarian cancer | | 25 | 19 |
| Basket 47: Gestational trophoblastic disease (GTD) | | 4 | 0 |
| Basket 48: Gallbladder cancer | | 22 | 19 |
| Basket 49: Small cell carcinoma of the ovary, hypercalcemic type | | 7 | 5 |
| Basket 50: PD-L1 amplified tumors | | 13 | 5 |
| Basket 51: Angiosarcoma | | 18 | 18 |
| Basket 52: High-grade neuroendocrine carcinoma | | 21 | 20 |
| Basket 53: Treatment-emergent small-cell neuroendocrine prostate cancer (t-SCNC) | | 19 | 16 |

**Table S2:** Cox regression model summaries of overall survival by baseline tumor size quartile. As a sensitivity analysis to models presented in Figure 2A,B, histologic baskets (see Table S1) were analyzed with frailty models instead of with a stratified analysis. Multivariable models were complete case and controlled for baseline age, sex, and ECOG performance status (PS) regardless of univariate significance.

|  |  | **Progression-free survival** | | **Overall survival** | |
| --- | --- | --- | --- | --- | --- |
| **Baseline sum of target lesions quartile** | **N (%)** | **Univariate**  Hazard ratio (95% confidence interval) p-value | **Multivariable**  Hazard ratio (95% confidence interval) p-value | **Univariate**  Hazard ratio (95% confidence interval) p-value | **Multivariable**  Hazard ratio (95% confidence interval) p-value |
| Quartile 1:  1.0-4.8cm | 188 (26) | Reference | Reference | Reference | Reference |
| Quartile 2:  4.8-8.0cm | 175 (24) | 1.10 (0.88-1.37)  0.40 | 1.07 (0.86-1.34)  0.54 | 1.15 (0.90-1.46)  0.28 | 1.13 (0.89-1.44)  0.33 |
| Quartile 3:  8.1-12.8cm | 180 (25) | 1.00 (0.80-1.25)  >0.99 | 1.09 (0.78-1.23)  **0.87** | 1.33 (1.05-1.69)  **0.020** | 1.32 (1.04-1.69)  **0.023** |
| Quartile 4:  12.9+cm | 178 (25) | 1.41 (1.12-1.77)  **0.003** | 1.33 (1.05-1.67)  **0.016** | 1.80 (1.41-2.30)  **<0.001** | 1.68 (1.31-2.15)  **<0.001** |

**Table S3:** Multivariable Cox regression summaries of overall survival by number of quartiles of baseline sum of target lesions; landmark analysis among patients alive on day 65. Cox models were stratified by histologic basket. Multivariable models were complete case and controlled for baseline age, sex, and ECOG performance status (PS) regardless of univariate significance.

|  | **N (%)** | **Univariate**  Hazard ratio (95% confidence interval) p-value | **Multivariable**  Hazard ratio (95% confidence interval) p-value | **Interpretation** |
| --- | --- | --- | --- | --- |
| **Baseline sum of target lesions quartile** |  |  |  |  |
| Quartile 1:  1.0-4.8cm | 164 (26) | Reference | Reference |  |
| Quartile 2:  4.8-8.0cm | 163 (26) | 1.30 (0.98-1.72)  0.066 | 1.31 (0.99-1.74)  0.060 | There was no significant difference in OS between the 1^st^ and 2^nd^ quartiles |
| Quartile 3:  8.1-12.8cm | 161 (25) | 1.54 (1.16-2.05)  **0.003** | 1.55 (1.16-2.06)  **0.003** | OS was significantly worse in the 3^rd^ quartile compared to the 1^st^ quartile |
| Quartile 4:  12.9+cm | 148 (23) | 1.78 (1.32-2.38)  **<0.001** | 1.72 (1.27-2.34)  **<0.001** | OS was significantly worse in the 4^th^ quartile compared to the 1^st^ quartile |
| **Tumor regression at first on-treatment scan** |  |  |  |  |
| No tumor regression | 458 (73) | Reference | Reference |  |
| Any tumor regression | 167 (27) | 0.36 (0.38-0.56)  **<0.001** | 0.34 (0.26-0.43)  **<0.001** | OS was significantly longer with any tumor regression compared to no tumor regression |

**Table S4:** Multivariable Cox regression summaries of overall survival by number of target lesions; landmark analysis among patients alive on day 65. Cox models were stratified by histologic basket. Multivariable models were complete case and controlled for baseline age, sex, and ECOG performance status (PS) regardless of univariate significance.

|  | **N (%)** | **Univariate**  Hazard ratio (95% confidence interval) p-value | **Multivariable**  Hazard ratio (95% confidence interval) p-value | **Interpretation** |
| --- | --- | --- | --- | --- |
| **Number of target lesions** |  |  |  |  |
| 1 target lesion | 130 (20) | Reference | Reference |  |
| 2 target lesions | 216 (34) | 0.99 (0.74-1.32)  0.95 | - 1. (0.75-1.34)   0.96 | There was no significant difference in OS between 1 and 2 target lesions |
| 3 target lesions | 128 (20) | 1.33 (0.97-1.82)  0.080 | 1.31 (0.95-1.80)  0.10 | There was no significant difference in OS between 1 and 3 target lesions |
| 4 target lesions | 98 (15) | 1.51 (1.08-2.11)  **0.017** | 1.50 (1.07-2.11)  **0.018** | OS was significantly worse in with 4 target lesions versus 1 target lesion |
| 5 target lesions | 64 (10) | 1.82 (1.24-2.66)  **0.002** | 1.77 (1.21-2.61)  **0.003** | OS was significantly worse in with 5 target lesions versus 1 target lesions |
| **Tumor regression at first on-treatment scan** |  |  |  |  |
| No tumor regression | 458 (73) | Reference | Reference |  |
| Any tumor regression | 167 (27) | 0.36 (0.28-0.46)  **<0.001** | 0.35 (0.27-0.44)  **<0.001** | OS was significantly longer with any tumor regression compared to no tumor regression |

**Figure S1A:** Kaplan-Meier estimates and Cox regression model summaries of progression-free survival by number of target lesions. Cox models were stratified by histologic basket. Multivariable models were complete case and controlled for baseline age, sex, and ECOG performance status (PS) regardless of univariate significance.

| **Number of target lesions** | **N (%)** | **Univariate**  Hazard ratio (95% confidence interval) p-value | **Multivariable**  Hazard ratio (95% confidence interval) p-value | **Interpretation** |
| --- | --- | --- | --- | --- |
| 1 | 148 (20) | Reference | Reference |  |
| 2 | 241 (33) | 0.98 (0.77-1.24)  0.86 | 0.98 (0.77-1.25)  0.87 | There was no significant difference in PFS between 1 and 2 target lesions |
| 3 | 140 (19) | 1.00 (0.77-1.31)  0.99 | 0.98 (0.75-1.28)  0.87 | There was no significant difference in PFS between 1 and 3 target lesions |
| 4 | 115 (26) | 1.38 (1.04-1.84)  **0.026** | 1.36 (1.02-1.82)  **0.033** | PFS was significantly worse in with 4 target lesions versus 1 target lesions |
| 5 | 78 (11) | 1.33 (0.98-1.82)  0.070 | 1.35 (0.99-1.85)  0.059 | There was no significant difference in PFS between 1 and 5 target lesions |

**Figure S1B:** Kaplan-Meier estimates and Cox regression model summaries of overall survival by number of target lesions. Cox models were stratified by histologic basket. Multivariable models were complete case and controlled for baseline age, sex, and ECOG performance status (PS) regardless of univariate significance.

| **Number of target lesions** | **N (%)** | **Univariate**  Hazard ratio (95% confidence interval) p-value | **Multivariable**  Hazard ratio (95% confidence interval) p-value | **Interpretation** |
| --- | --- | --- | --- | --- |
| 1 | 148 (20) | Reference | Reference |  |
| 2 | 241 (33) | 0.98 (0.77-1.24)  0.86 | 0.92 (0.71-1.19)  0.53 | There was no significant difference in OS between 1 and 2 target lesions |
| 3 | 140 (19) | 1.00 (0.77-1.31)  0.99 | 1.13 (0.85-1.50)  0.39 | There was no significant difference in OS between 1 and 3 target lesions |
| 4 | 115 (26) | 1.38 (1.04-1.84)  **0.026** | 1.52 (1.13-2.04)  **<0.001** | OS was significantly worse in with 4 target lesions versus 1 target lesion |
| 5 | 78 (11) | 1.33 (0.98-1.82)  0.070 | 1.78 (1.27-2.48)  **<0.001** | OS was significantly worse in with 5 target lesions versus 1 target lesion in multivariable but not univariate analysis |
